# Supplementary material for: Parent-reported phenotype data on chromosome 6 aberrations collected via an online questionnaire: data consistency and data availability
Source: Orphanet J Rare Dis. 2023 Mar 19;18:60. doi: 10.1186/s13023-023-02657-x (PMC10024830; doi:10.1186/s13023-023-02657-x)
Supplement: Supplementary file 1 — Additional file 1: Supplementary Information S1. List of included questions of Chromosome 6 Questionnaire. Table S1. Country of origin of invited and test panel parents. Table S2. Number of pages collected from medical files. Table S3. Number of health professionals from who medical files were received. Table S4. Number of follow-up measurements for growth available per individual. Table S5. Consistency main questions. Table S6. Consistency sub-questions. Table S7. Number of answers unknown from medical files data. Table S8. Type of inconsistency per question category. Table S9. Type and number of inconsistencies per test panel parent. Table S10. Number of questions for which the availability of data was significantly, or not significantly different when collected from parents compared to literature. Table S11. All p-values main questions. Table S12. All p-values sub-questions. Fig. S1. Flowchart inclusion individuals data availability study. Fig. S2. Total answers checked for consistency and distribution of consistent and inconsistent answers per test parent. [file 13023_2023_2657_MOESM1_ESM.pdf]

Additional File 1: Supplementary files

Parent-reported phenotype data on chromosome 6 aberrations collected via an online questionnaire: data consistency and data availability

Aafke Engwerda, Barbara Frentz, Eleana Rraku, Nadia F. Simoes de Souza, Morris A. Swertz, Mirjam Plantinga, Wilhelmina S. Kerstjens-Frederikse, Adelita V. Ranchor and Conny M. A. van Ravenswaaij-Arts

Supplementary Tables

Table S1. Country of origin of invited and test panel parents
Table S2. Number of pages collected from medical files
Table S3. Number of health professionals from who medical files were received
Table S4. Number of follow-up measurements for growth available per individual
Table S5. Consistency main questions
Table S6. Consistency sub-questions
Table S7. Number of answers unknown from medical files data
Table S8. Type of inconsistency per question category
Table S9. Type and number of inconsistencies per test panel parent
Table S10. Number of questions for which the availability of data was significantly, or not significantly different when collected from parents compared to literature
Table S11. All p-values main questions
Table S12. All p-values sub-questions

Supplementary Figures

Figure S1. Flowchart inclusion individuals data availability study
Figure S2. Total answers checked for consistency and distribution of consistent and inconsistent answers per test parent

Supplementary Information

Supplementary Information S1. List of included questions of Chromosome 6 Questionnaire

Table S1. Country of origin of invited and test panel parents

Country of origin	Number of parents invited	Number of parents in test panel
United Kingdom	7	4
Ireland	1	1
United States of America	28	11
Canada	2	1
Australia	4	2
New Zealand	1	1

Table S2. Number of pages collected from medical files

Total number of pages received per patient median (range)	Number of health professionals per patient median (range)	Average number of pages per health professional per patient median (range)
47.5 (15–121)	4 (2–11)	10.4 (3.4–43)

Table S3. Number of health professionals from whom medical files were received

Health professionals from whom medical files were received	Number of individuals for whom medical files were received
Geneticist	18
Neurologist	14
Paediatrician	10
General Practitioner	9
Ophthalmologist	9
Otorhinolaryngologist	5
Speech therapist	4
Gastroenterologist	4
Cardiologist	3
Occupational therapist	3
Orthopaedics	3
Physiotherapist	2
Pulmonologist	2
Neurosurgeon	1
Endocrinologist	1
Psychologist	1
Paediatric Surgeon	1
General surgeon	1
Infectious Disease specialist	1
Audiologist	1

Information from the General Practitioner often also included letters from other health professionals not mentioned in this table, for example trauma surgeons, plastic surgeons, metabolic specialists and development specialists.

Table S4. Number of follow-up measurements for growth available per individual

Follow-up measurements	Parent data Median (range)	Medical file data Median (range)
Height	3.5 (0–5)	1.5 (1–4)
Weight	4 (0–5)	2 (1–4)
Head circumference	1.5 (0–5)	1 (0–3)

Table S5. Consistency main questions

	Consistency main questions			Answer unknown	
	Total answers	Consistent/observed (+ probably consistent/observed)*	Consistency – maximum consistency*	Parent data	Medical files data
**Congenital malformations**	**740**	**130/167 (627/664)**	**78–94%**	**3**	**570**
Physical aspects	580	89/115 (492/518)	77–95%	3	462
Head	100	29/29 (91/91)	100%	3	68
Face	160	20/26 (131/137)	77–96%	0	134
Trunk	180	23/31 (166/174)	74–95%	0	149
Limbs	80	14/21 (54/61)	67%–89%	0	59
Skin	60	3/8 (50/55)	38–91%	0	52
Heart and vessels	40	9/10 (37/38)	90–97%	0	30
Airways and lungs	60	6/8 (49/51)	75–96%	0	52
Kidneys and genitals	40	14/17 (34/37)	82–92%	0	23
Brain	20	12/17 (15/20)	71–75%	0	3
**Medical problems**	**744**	**146/174 (648/676)**	**84–96%**	**0**	**570**
Senses	100	36/48 (79/91)	75–87%	0	52
Visual	60	25/35 (47/57)	71–82%	0	25
Hearing	40	11/13 (32/34)	85–94%	0	27
Feeding and the gut	160	27/31 (142/146)	87–97%	0	129
Feeding	20	9/9 (13/13)	100%	0	11
Gut	140	18/22 (129/133)	82–97%	0	118
Heart and vessels	40	1/1 (39/39)	100%	0	39
Blood, hormones and immune system	204	24/28 (184/188)	86–98%	0	176
Blood	60	4/5 (57/58)	80–98%	0	55
Hormones[&]	104	15/17 (101/103)	88–98%	0	87
Immune system	40	5/6 (26/27)	83–96%	0	34
Neurology and Epilepsy	240	58/66 (204/212)	88–96%	0	174
Neurology	220	47/55 (186/194)	85–96%	0	165
Epilepsy	20	11/11 (18/18)	100%	0	9
**Behaviour**	**60**	**8/13 (45/50)**	**62–90%**	**0**	**47**
Diagnosis	40	4/6 (32/34)	67–94%	0	34
Sleeping	20	4/7 (13/16)	57–81%	0	13
**Growth measurements**	**81**	**50/53 (na)**	**94%**	**1**	**27**
Birth measurements	48	19/20 (na)	95%	1	27
Birth weight	19	9/9 (na)	100%	1	9
Birth length	18	8/8 (na)	100%	0	10
Head circumference birth	11	2/3 (na)	67%	0	8
Follow-up measurements	33	31/33 (na)	94%	na	na
Height	na	12/13 (na)	92%	na	na
Weight	na	12/13 (na)	92%	na	na
Head circumference	na	7/7 (na)	100%	na	na
**Development**	**438**	**109/114 (192/197)**	**96–97%**	**2**	**322**
IQ	40	21/23 (27/29)	91–93%	0	17
Milestones	398	88/91 (165/168)	97–98%	2	305
Gross motor	160	52/52 (61/61)	100%	0	108
Fine motor	100	4/5 (39/40)	80–98%	0	95
Communication	100	30/32 (43/45)	94–96%	0	68
Toilet training[#]	38	2/2 (22/22)	100%	2	34

**Total**	**2063**	**443/521 (1562/1640)**	**85–95%**	**6**	**1536**

*Including the probably consistent answers, see Methods.

&For 16 individuals, the question about puberty was not applicable due to young age

#For 1 individual, the questions about toilet training milestones were not completed.

na = not applicable

Table S6. Consistency sub questions

	**Consistency sub questions**			Answer unknown	
	Total answers	consistent/observed (+ partly consistent/observed)*	% consistency – maximum consistency*	Parent data	Medical file data
**Congenital malformations**	**85**	**67/79 (79/79)**	**85–100%**	**0**	**6**
Physical aspects	51	45/47 (47/47)	96–100%	0	4
Head	7	7/7	100%	0	0
Face	10	9/10 (10/10)	90–100%	0	0
Trunk	8	8/8	100%	0	0
Limbs	19	16/16	100%	0	3
Skin	7	5/6 (6/6)	83–100%	0	1
Heart and vessels	3	2/3 (3/3)	67–100%	0	0
Airways and lungs	11	10/11 (11/11)	91–100%	0	0
Kidneys and genitals	8	6/7 (7/7)	86–100%	0	1
Brain	12	4/11 (11/11)	36–100%	0	1
**Medical problems**	**115**	**49/78 (75/78)**	**63–96%**	**1**	**36**
Senses	38	16/22 (22/22)	73–100%	1	15
Visual	18	11/17 (17/17)	65–100%	1	0
Hearing	20	5/5	100%	0	15
Feeding and the gut	21	9/21 (21/21)	43–100%	0	0
Feeding	12	3/12 (12/12)	25–100%	0	0
Gut	9	6/9 (9/9)	67–100%	0	0
Heart and vessels	0	0/0		0	0
Blood, hormones and immune system	2	2/2	100%	0	0
Blood	0	0/0		0	0
*Hormones**	na	na	na	na	na
Immune system	2	2/2	100%	0	0
Neurology and epilepsy	54	22/33 (30/33)	67–91%	0	21
Neurology	na	na	na	na	na
Epilepsy	54	22/33 (30/33)	67–91%	0	21
**Behaviour**	**6**	**4/5 (5/5)**	**80–100%**	**1**	**0**
Diagnosis	3	3/3	100%	0	0
Sleeping	3	1/2 (2/2)	50–100%	1	0
**Development**	**89**	**36/41**	**88%**	**19**	**29**
IQ	9	1/1	100%	4	4
Milestone	80	35/40	88%	15	25
Gross motor	58	30/34	88%	9	15
Fine motor	0	0/0		0	0
Communication	20	5/6	83%	4	10
Toilet training	2	0/0		2	0
**Total**	**295**	**156/203 (195/203)**	**77–96%**	21	71

na = not applicable, this subcategory did not contain sub-questions.

*including the partly consistent answers, see Methods

Table S7. Number of answers unknown from medical files data

	**Number of unknown answers for which parents reported absent**	**Number of unknown answers for which parents reported present**
Congenital malformations	497	73
Medical problems	502	68
Behaviour	37	10
Development	83	239
**Total**	**1119**	**390**

Table S8. Type of inconsistency per question category

	Major/total answers	Minor/partly/total answers
**Congenital malformations**	**37/167**	**0/12/79**
Airways and lungs	2/8	0/1/11
Brain	5/17	0/7/11
Heart and vessels	1/10	0/1/3
Kidneys and genitals	3/17	0/1/7
Physical aspects	26/115	0/2/47
Face	6/26	0/1/10
Head	0/29	0/0/7
Limbs	7/21	0/0/16
Skin	5/8	0/1/6
Trunk	8/31	0/0/8
**Medical problems**	**21/142**	**10/26/110**
Blood, hormones and immune system	4/28	0/0/2
Blood	1/5	0/0/0
Hormones	2/17	na
Immune system	1/6	0/0/2
Epilepsy and neurology	1/34	10/8/65
Epilepsy	0/11	3/8/33
Neurology	1/23	7/0/32
Feeding and the gut	4/31	0/12/21
Feeding	0/9	0/9/12
Gut	4/22	0/3/9
Heart and vessels	0/1	0/0/0
Senses	12/48	0/6/22
Hearing	2/13	0/0/5
Visual	10/35	0/6/17
**Growth measurements**	**3/53**	**na**
Birth measurements	1/20	na
Birth weight	0/9	na
Birth length	0/8	na
Head circumference birth	1/3	na
Follow-up measurements	2/33	na
Height	1/13	na
Weight	1/13	na
Head circumference	0/7	na
**Behaviour**	**5/13**	**0/1/5**
Diagnosis	2/6	0/0/3
Sleeping	3/7	0/1/2
**Development**	**5/114**	**5/na/41**
IQ test	2/23	0/na/1
Milestone	3/91	5/na/40
Gross motor	0/52	4/na/34
Fine motor	1/5	0/na/0
Communication	2/32	1/na/6
Toilet training	0/2	0/na/0
**Total**	**71/489**	**15/39/235**

Table S9. Type and number of inconsistencies per test panel parent

	Consistent/ observed	consistency	Total inconsistent	Major	Minor	Partly
**1**	39/49	80%	10	6	1	3
**2**	41/51	80%	10	8	1	1
**3**	28/31	90%	3	3	0	0
**4**	39/48	81%	9	2	0	7
**5**	28/37	76%	9	4	1	4
**6**	14/17	82%	3	3	0	0
**7**	20/20	100%	0	0	0	0
**8**	31/35	89%	4	1	0	3
**9**	30/33	91%	3	3	0	0
**10**	18/22	82%	4	2	1	1
**11**	24/26	92%	2	2	0	0
**12**	36/42	86%	6	4	2	0
**13**	23/31	74%	8	2	2	4
**14**	36/41	88%	5	2	0	3
**15**	36/42	86%	6	5	0	1
**16**	32/42	76%	10	5	3	2
**17**	44/59	75%	15	8	3	4
**18**	27/32	84%	5	1	0	4
**19**	20/28	71%	8	8	0	0
**20**	33/38	87%	5	2	1	2

This data is visualised in Figure 2 and Figure S3.

Table S10. Number of questions for which the availability of data was significantly (or not significantly) different when collected from parents compared to literature

	Main questions	Sub-questions
	**Significant more data from parents/ significant more data from literature/ no significant difference (α=0.05)**	**Significant more data from parents/ significant more data from literature/ no significant difference (α=0.05)**
**Congenital malformations**	**37/0/1**	**1/1/24**
Airways and lungs	3/0/0	0/0/3
Brain	0/0/1	0/1/0
Heart and vessels	2/0/0	0/0/2
Kidneys and genitals	3/0/0	0/0/3
Physical aspects	29/0/0	1/0/16
Face	8/0/0	0/0/3
Head	5/0/0	0/0/2
Limbs	4/0/0	1/0/3
Skin	3/0/0	0/0/3
Trunk	9/0/0	0/0/5
**Medical problems**	**38/0/0**	**2/0/19**
Blood, hormones and immune system	11/0/0	0/0/4
Blood	3/0/0	0/0/3
Hormones	6/0/0	na
Immune system	2/0/0	0/0/1
Epilepsy and neurology	12/0/0	1/0/3
Epilepsy	1/0/0	1/0/3
Neurology	11/0/0	na
Feeding and the gut	8/0/0	1/0/5
Feeding	1/0/0	1/0/1
Gut	7/0/0	0/0/4
Heart and vessels	2/0/0	0/0/2
Senses	5/0/0	0/0/5
Hearing	2/0/0	0/0/2
Visual	3/0/0	0/0/3
**Behaviour**	**3/0/0**	**0/0/3**
Diagnosis	2/0/0	0/0/2
Sleeping	1/0/0	0/0/1
**Growth measurements**	**6/0/0**	**na**
Birth measurements	3/0/0	na
Follow-up measurements	3/0/0	na
**Development**	**29/0/1**	**2/0/19**
IQ	0/0/2	0/0/1
Milestone	20/0/0	2/0/18
Gross motor	8/0/0	0/0/8
Fine motor	5/0/0	0/0/5
Communication	5/0/0	2/0/3
Toilet training	2/0/0	0/0/2
Adult functioning	8/0/0	na
**Total**	**113/0/2**	**5/1/65**

na= not applicable

Table S11. All p-values main questions

Main question	Parent known/total	Literature known/total	Significance* (α=0.05)
An abnormal head size	34/34	18/39	**<0.0001**
Abnormality of the fontanelles	24/34	1/39	**<0.0001**
Plagiocephaly (asymmetric skull)	32/34	1/39	**<0.0001**
Craniosynostosis (too early closure of the sutures of the skull)	30/34	0/39	**<0.0001**
Encephalocele (skull defect with protrusion of the brain and/or membranes)	21/34	0/39	**<0.0001**
Hypertelorism (widely spaced eyes)	32/34	19/39	**<0.0001**
Ptosis (drooping) of the upper eyelid	33/34	2/39	**<0.0001**
Blocked or absent tear ducts (nasolacrimal ducts)	31/34	1/39	**<0.0001**
An abnormal lip or palate	34/34	15/39	**<0.0001**
Dental (tooth) abnormalities	34/34	3/39	**<0.0001**
Skin pit or skin tag near the ears	33/34	0/39	**<0.0001**
Abnormality of the outer ear (malformed or dysplastic)	34/34	19/39	**<0.0001**
Abnormality of the auditory canal	32/34	1/39	**<0.0001**
Underdeveloped or absent collarbones (clavicles)	30/34	1/39	**<0.0001**
Abnormality of the ribs	30/34	3/39	**<0.0001**
An abnormal shape of the thorax	30/34	3/39	**<0.0001**
Extra nipples	30/34	0/39	**<0.0001**
An abdominal wall defect	34/34	2/39	**<0.0001**
Abnormal curvature of the back/spine	32/34	5/39	**<0.0001**
Abnormal vertebrae	30/34	3/39	**<0.0001**
Spina bifida	31/34	2/39	**<0.0001**
A sacral dimple (small depression in the skin, located just above the buttocks)	33/34	2/39	**<0.0001**
Hypermobility of the joints	34/34	14/39	**<0.0001**
Contractures of the joints (limited movement of joints due to fixation)	30/34	0/39	**<0.0001**
Particular features of the hands or fingers	34/34	4/39	**<0.0001**
Particular features of the feet or toes	34/34	8/39	**<0.0001**
Abnormalities of the skin	32/34	5/39	**<0.0001**
Abnormal hair	30/34	2/39	**<0.0001**

Abnormalities of the nails	34/34	1/39	**<0.0001**
Is there a congenital heart defect	34/34	10/39	**<0.0001**
Does your child have cardiomyopathy	34/34	6/39	**<0.0001**
Are there any nasal problems	33/34	0/39	**<0.0001**
Does your child have breathing, airway or lung problems	34/34	1/39	**<0.0001**
Does your child have a diaphragmatic problem	34/34	0/39	**<0.0001**
Does your child have renal or urinary tract problems	34/34	4/39	**<0.0001**
Does your son have abnormalities of the genitals	24/34	5/39	**<0.0001**
Does your daughter have abnormalities of the genitals	27/34	1/39	**<0.0001**
Were there abnormalities of the brain seen on imaging (MRI, CT, ultrasound)	27/34	36/39	0.1099 (0.1725)
Does your child have any problems with his/her eyes	33/34	12/39	**<0.0001**
Does your child have abnormal (involuntary) eye movements	34/34	19/39	**<0.0001**
Are there special characteristics of the eyes (cataract, coloboma, retinitis pigmentosa, glaucoma, anophthalmia, etc.)	34/34	7/39	**<0.0001**
Are there congenital abnormalities of the middle and/or inner ear	32/34	2/39	**<0.0001**
Does your child have a hearing impairment	34/34	4/39	**<0.0001**
Has your child had any problems in eating, drinking or swallowing	34/34	5/39	**<0.0001**
Oesophageal atresia (congenitally interrupted oesophagus)	34/34	1/39	**<0.0001**
A tracheo-oesophageal fistula (abnormal connection (fistula) between the oesophagus and the trachea)	34/34	1/39	**<0.0001**
Gastro-oesophageal or acid reflux (GERD)	34/34	0/39	**<0.0001**
An abnormality of the liver or poor liver function	34/34	0/39	**<0.0001**
An abnormality of the gallbladder or bile ducts	34/34	0/39	**<0.0001**
Bowel or defecation problems	34/34	1/39	**<0.0001**
Anal problems	32/34	1/39	**<0.0001**
Does your child have arrhythmia	31/34	4/39	**<0.0001**
Does your child have abnormal blood pressure	30/34	0/39	**<0.0001**
Are there abnormalities of the number of blood cells	30/34	0/39	**<0.0001**
Are there abnormalities in blood clotting	30/34	0/39	**<0.0001**
Is there an abnormality of the spleen	30/34	0/39	**<0.0001**
Growth hormone (too little/too much/no problem)	34/34	1/39	**<0.0001**
Thyroid hormone (too little/too much/no problem)	34/34	0/39	**<0.0001**
Adrenal hormones (too little/too much/no problem)	34/34	0/39	**<0.0001**
Diabetes mellitus	34/34	0/39	**<0.0001**

Diabetes insipidus (condition characterised by excessive thirst and excretion of large amounts of severely dilute urine)	30/34	0/39	**<0.0001**
Did your child enter puberty spontaneously	7/34	1/39	**0.0139 (0.0215)**
Does or did your child have recurrent infections	30/34	1/39	**<0.0001**
Does your child have a problem with his/her immune system	30/34	0/39	**<0.0001**
Facial palsy	30/34	0/39	**<0.0001**
Hypotonia or low muscle tone	32/34	28/39	**0.0129**
Hypertonia or excessive muscle tone	27/34	2/39	**<0.0001**
Spasticity (combination of paralysis, increased tendon reflex activity, hypertonia, excessive muscle contraction)	30/34	0/39	**<0.0001**
Hyperreflexia (overactive or over-responsive tendon reflexes)	30/34	2/39	**<0.0001**
Areflexia/hyporeflexia (below normal or absent tendon reflexes)	30/34	2/39	**<0.0001**
Myopathy (muscular disease resulting in muscular weakness)	30/34	1/39	**<0.0001**
Ataxia (poor coordination of muscle movements)	30/34	2/39	**<0.0001**
Torticollis (twisted neck due to hypertonia of neck muscles)	31/34	0/39	**<0.0001**
Is there an abnormality of the spinal cord (tethered cord)	34/34	8/39	**<0.0001**
Does your child have migraine headaches	30/34	0/39	**<0.0001**
Has your child ever had convulsions, fits or seizures	34/34	31/39	**0.0051 (0.0059)**
Was there a behavioural diagnosis made by a (child) psychiatrist or psychologist?	30/34	3/39	**0.0001**
Does your child have a mood disorder	30/34	0/39	**<0.0001**
Does your child have problems with sleeping	33/34	2/39	**<0.0001**
Weight at birth	33/34	11/39	**<0.0001**
Length at birth	27/34	10/39	**<0.0001**
Head circumference at birth	18/34	10/39	**0.0167 (0.0291)**
Height follow-up	31/34	12/39	**<0.0001**
Weight follow-up	33/34	10/39	**<0.0001**
Head circumference follow-up	29/34	22/39	**0.0073 (0.0102)**
Is there developmental delay	25/25	31/31	1 (1)
Has an IQ, DQ or developmental test been done	34/34	6/39	**<0.0001**
Reach for objects	33/34	0/39	**<0.0001**
Roll over from lying on back to lying on tummy	34/34	1/39	**<0.0001**
Sit up unsupported	34/34	3/39	**<0.0001**
Get to a sitting position without help	34/34	5/39	**<0.0001**
Crawl or bottom shuffle	34/34	1/39	**<0.0001**

Pull him- or herself up a standing position	34/34	1/39	**<0.0001**
Walk holding on to furniture (e.g. to a table)	34/34	1/39	**<0.0001**
Walk unsupported	34/34	14/39	**<0.0001**
Pick up something, like an object the size of a currant, between thumb and index finger	33/34	0/39	**<0.0001**
Hold a pencil between thumb and index finger	32/34	0/39	**<0.0001**
Colour within the lines	32/34	0/39	**<0.0001**
Build a tower of 6 blocks	34/34	0/39	**<0.0001**
String beads	34/34	0/39	**<0.0001**
Smile	34/34	0/39	**<0.0001**
Make sounds; cooing, gurgle	33/34	1/39	**<0.0001**
Make sounds; babble, copy sounds he or she hears	33/34	2/39	**<0.0001**
Speak his or her first words	34/34	7/39	**<0.0001**
Speak 2-word sentences	34/34	7/39	**<0.0001**
Is your child fully toilet trained (urine and defecation) during the day	34/34	2/39	**<0.0001**
Is your child fully toilet trained (urine and defecation) during the night	34/34	1/39	**<0.0001**
Ability to use telephone[#]	2/2	0/8	**0.0222**
Shopping[#]	2/2	0/8	**0.0222**
Food preparation[#]	2/2	0/8	**0.0222**
Housekeeping[#]	2/2	0/8	**0.0222**
Laundry[#]	2/2	0/8	**0.0222**
Mode of transportation[#]	2/2	0/8	**0.0222**
Responsibility for own medications[#]	2/2	0/8	**0.0222**
Ability to handle finances[#]	2/2	0/8	**0.0222**

[#] Questions only applicable to adults (>18 years)

* Assessed using Chi-square (Fisher's exact test). A Fisher's exact test was performed when applicable, 20% of the expected counts <5

Bold text indicates significant outcomes

Table S12. All p-values sub questions

Main question followed by sub question	Parent known/main question present	Literature known/main question present	Significance* (α=0.05)
An abnormal head size → What kind (microcephaly/macrocephaly)	15/15	16/16	1
Craniosynostosis (too early closure of sutures of the skull) → Which suture(s)	1/1	0/0	1
An abnormal lip or palate → Please specify	2/7	0/13	0.1105
Dental (tooth) abnormalities → Please specify	7/10	1/3	0.5105
Abnormality of the auditory canal → Please specify	2/3	0/0	1
Abnormality of the ribs → Please specify	0/0	1/2	1
An abnormal shape of the thorax → Please specify	0/0	1/2	1
An abdominal wall defect → Please specify	4/4	2/2	1
Abnormal curvature of the back/spine → Please specify	3/3	3/3	1
Abnormal vertebrae → Please specify	3/3	0/1	0.25
Hypermobility of the joints → Please specify	15/16	2/14	**<0.0001**
Contractures of the joints (limited movement of joints due to fixation) → Please specify	2/4	0/0	1
Particular features of the hands or fingers → Please specify	2/7	1/3	1
Particular features of the feet or toes → Please specify	5/6	7/7	0.4615
Abnormalities of the skin → Please specify	6/8	0/4	0.0606
Abnormal hair → Please specify	0/0	1/1	1
Abnormalities of the nails → Please specify	0/0	0/1	1
Is there a congenital heart defect → Please specify	8/8	3/4	0.3333
Does your child have cardiomyopathy → Please specify	1/1	0/0	1
Are there any nasal problems → Please specify	1/2	0/0	1
Does your child have breathing, airway or lung problems → Please specify	5/7	0/1	0.375
Does your child have a diaphragmatic problem → Please specify	2/2	0/0	1
Does your child have renal or urinary tract problems → Please specify	4/5	1/1	1
Does your son have abnormalities of the genitals → Please specify	0/2	1/2	1
Does your daughter have abnormalities of the genitals → Please specify	3/5	0/1	1
Were there abnormalities of the brain seen on imaging (MRI, CT, ultrasound) → Please specify	16/22	35/35	**0.0021**
Does your child have any problems with his/her eyes → Please specify	20/24	5/6	1

Does your child have abnormal (involuntary) eye movements → Please specify	14/16	13/14	1
Are there special characteristics of the eyes (cataract, coloboma, retinitis pigmentosa, glaucoma, anophthalmia, etc.) → Please specify	4/9	1/2	1
Are there congenital abnormalities of the middle and/or inner ear → Please specify	0/4	0/0	1
Does your child have a hearing impairment → Is there progressive hearing loss	0/0	0/0	no data
Has your child had any problems in eating, drinking or swallowing→ Please specify	23/25	0/3	**0.0031**
Has your child had any problems in eating, drinking or swallowing → Was one or both of the following needed for feeding problems (tube feeding)	7/25	2/3	0.2344
An abnormality of the liver or poor liver function → Please specify	0/1	0/0	1
An abnormality of the gallbladder or bile ducts → Please specify	0/0	0/0	no data
Bowel or defecation problems → Please specify	14/14	1/1	1
Anal problems → Please specify	3/4	1/1	1
Does your child have arrhythmia → Please specify	0/0	0/0	no data
Does your child have abnormal blood pressure → Please specify	1/2	0/0	1
Are there abnormalities of the number of blood cells → Please specify	1/1	0/0	1
Are there abnormalities in blood clotting → Please specify	1/1	0/0	1
Is there an abnormality of the spleen → Please specify	0/0	0/0	no data
Does your child have a problem with his/her immune system → Please specify	0/2	0/0	1
Has your child ever had convulsions, fits or seizures → What kind of seizures	17/19	17/24	0.2575
Has your child ever had convulsions, fits or seizures → Was epilepsy formally diagnosed	13/19	15/24	0.7554
Has your child ever had convulsions, fits or seizures → Is or was there medication for epilepsy used	17/19	9/24	**0.0006**
Has your child ever had convulsions, fits or seizures → Has your child ever had a status epilepticus (an epileptic seizure of more than 30 minutes)	3/19	1/24	0.306
Was there a behavioural diagnosis made by a (child) psychiatrist or psychologist? → Please specify	6/7	3/3	1
Does your child have a mood disorder → Please specify	2/2	0/0	1
Does your child have problems with sleeping → What kind of sleeping problems	13/15	1/2	0.3309
Has an IQ, DQ or developmental test been done → What was the result of the testing	10/10	6/6	1
Reach for objects → Since what age (months)	28/32	0/0	1
Roll over from lying on back to lying on tummy → Since what age (months)	29/32	1/1	1
Sit up unsupported → Since what age (months)	30/31	2/2	1
Sit up unsupported → Since what age (months)	25/30	4/4	1
Crawl or bottom shuffle → Since what age (months)	27/29	1/1	1
Pull him- or herself up a standing position → Since what age (months)	24/25	0/0	1

Walk holding on to furniture (e.g. to a table) → Since what age (months)	22/23	0/0	1
Walk unsupported → Since what age (months)	18/19	10/12	0.5435
Pick up something, like an object the size of a currant, between thumb and index finger → Since what age (months)	21/27	0/0	1
Hold a pencil between thumb and index finger → Since what age (months)	11/15	0/0	1
Colour within the lines → Since what age (months)	4/15	0/0	1
Build a tower of 6 blocks → Since what age (months)	13/15	0/0	1
String beads → Since what age (months)	14/16	0/0	1
Smile → Since what age (weeks)	31/34	0/0	1
Make sounds; cooing, gurgle → Since what age (weeks)	24/31	0/1	1
Make sounds; babble, copy sounds he or she hears → Since what age (months)	25/30	1/1	1
Speak his or her first words → Since what age (months)	23/25	2/5	**0.0219**
Speak 2-word sentences → Since what age (months)	18/20	1/5	**0.0055**
Is your child fully toilet trained (urine and defecation) during the day → Since what age (years)	10/10	0/0	1
Is your child fully toilet trained (urine and defecation) during the night → Since what age (years)	7/7	0/0	1

* Assessed using Fisher's exact test

Bold text indicates significant outcomes. Sub-questions were only shown in the questionnaire when applicable (abnormality present or milestone achieved).

Figure S1. Flowchart inclusion of individuals for data availability study

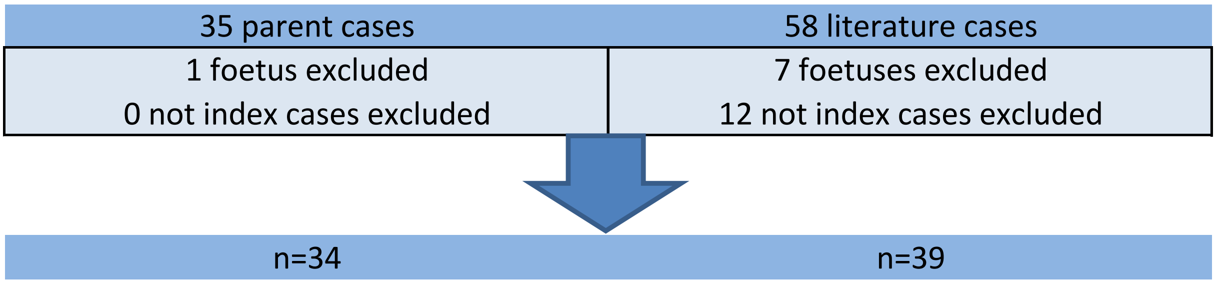

Figure S2. Total answers checked for consistency and distribution of consistent and inconsistent answers per test parent

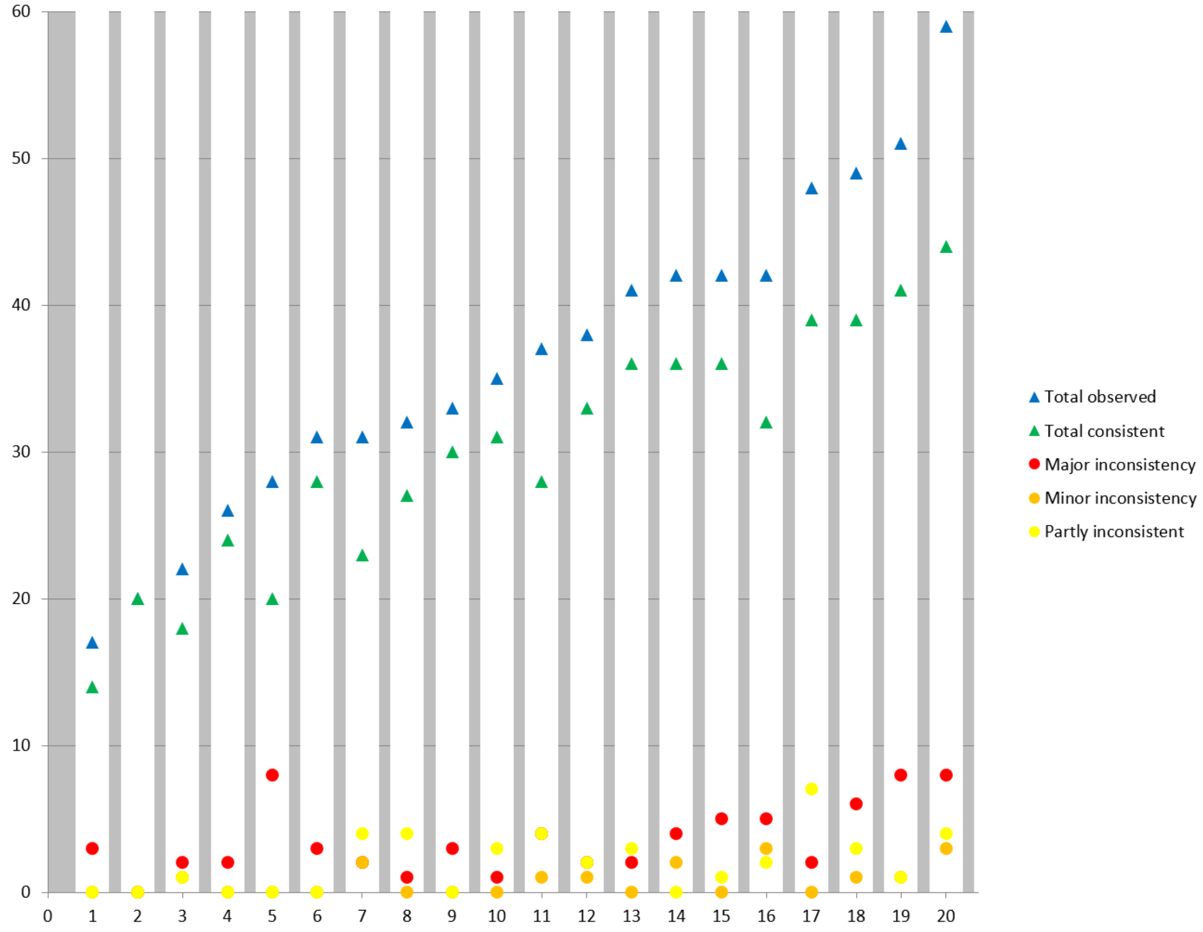

X-axis is the 20 test panel parents. Y-axis is the number of answers.

Supplementary Information S1. List of included questions of Chromosome 6 Questionnaire

All included main and sub questions. Sub questions are underlined. Categories and subcategories of questions are in bold.
* = Main question, but considered minor when answers were inconsistent

Congenital malformations

Physical aspects

Head

An abnormal head size

<u>What kind (microcephaly/macrocephaly)</u>

Abnormality of the fontanelles

Plagiocephaly (asymmetric skull)

Craniosynostosis (too early closure of sutures of the skull)

<u>Which suture(s)</u>

Encephalocele (skull defect with protrusion of the brain and/or membranes)

Face

Hypertelorism (widely spaced eyes)

Ptosis (drooping) of the upper eyelid

Blocked or absent tear ducts (nasolacrimal ducts)

An abnormal lip or palate

<u>Please specify</u>

Dental (tooth) abnormalities

<u>Please specify</u>

Skin pit or skin tag near the ears

Abnormality of the outer ear (malformed or dysplastic)

Abnormality of the auditory canal

<u>Please specify</u>

Trunk

Underdeveloped or absent collarbones (clavicles)

Abnormality of the ribs

<u>Please specify</u>

An abnormal shape of the thorax

<u>Please specify</u>

Extra nipples

An abdominal wall defect

<u>Please specify</u>

Abnormal curvature of the back/spine

<u>Please specify</u>

Abnormal vertebrae

<u>Please specify</u>

Spina bifida

A sacral dimple (small depression in the skin, located just above the buttocks)

Limbs
Hypermobility of the joints
Please specify
Contractures of the joints (limited movement of joints due to fixation)
Please specify
Particular features of the hands or fingers
Please specify
Particular features of the feet or toes
Please specify

Skin
Abnormalities of the skin
Please specify
Abnormal hair
Please specify
Abnormalities of the nails
Please specify

Heart and vessels
Is there a congenital heart defect
Please specify
Does your child have cardiomyopathy
Please specify

Airways and lungs
Are there any nasal problems
Please specify
Does your child have breathing, airway or lung problems
Please specify
Does your child have a diaphragmatic problem
Please specify

Kidneys and genitals
Does your child have renal or urinary tract problems
Please specify
Does your son have abnormalities of the genitals
Please specify
Does your daughter have abnormalities of the genitals
Please specify

Brain
Were there abnormalities of the brain seen on imaging (MRI, CT, ultrasound)
Please specify

Medical problems

Senses

Visual

Does your child have any problems with his/her eyes

Please specify

Does your child have abnormal (involuntary) eye movements

Please specify

Are there special characteristics of the eyes (cataract, coloboma, retinitis pigmentosa, glaucoma, anophthalmia, etc.)

Please specify

Hearing

Are there congenital abnormalities of the middle and/or inner ear

Please specify

Does your child have a hearing impairment

Is there progressive hearing loss

Feeding and the gut

Feeding

Has your child had any problems in eating, drinking or swallowing

Please specify

Was one or both of the following needed for feeding problems (tube feeding)

Gut

*Oesophageal atresia (congenitally interrupted oesophagus)

*A tracheo-oesophageal fistula (abnormal connection (fistula) between the oesophagus and the trachea)

Gastro-oesophageal or acid reflux (GERD)

An abnormality of the liver or poor liver function

Please specify

An abnormality of the gallbladder or bile ducts

Please specify

Bowel or defecation problems

Please specify

Anal problems

Please specify

Heart and vessels

Does your child have arrhythmia

Please specify

Does your child have abnormal blood pressure

Please specify

Blood, hormones and immune system

Blood

Are there abnormalities of the number of blood cells

<u>Please specify</u>

Are there abnormalities in blood clotting

<u>Please specify</u>

Is there an abnormality of the spleen

<u>Please specify</u>

Hormones

Growth hormone (too little/too much/no problem)

Thyroid hormone (too little/too much/no problem)

Adrenal hormones (too little/too much/no problem)

Diabetes mellitus

Diabetes insipidus (condition characterised by excessive thirst and excretion of large amounts of severely dilute urine)

Did your child enter puberty spontaneously

Immune system

Does or did your child have recurrent infections

Does your child have a problem with his/her immune system

<u>Please specify</u>

Neurology and epilepsy

Neurology

Facial palsy

*Hypotonia or low muscle tone

*Hypertonia or excessive muscle tone

*Spasticity (combination of paralysis, increased tendon reflex activity, hypertonia, excessive muscle contraction)

*Hyperreflexia (overactive or over-responsive tendon reflexes)

*Areflexia/hyporeflexia (below normal or absent tendon reflexes)

Myopathy (muscular disease resulting in muscular weakness)

Ataxia (poor coordination of muscle movements)

Torticollis (twisted neck due to hypertonia of neck muscles)

Is there an abnormality of the spinal cord (tethered cord)

Does your child have migraine headaches

Epilepsy

Has your child ever had convulsions, fits or seizures

<u>What kind of seizures</u>

<u>Was epilepsy formally diagnosed</u>

<u>Is or was there medication for epilepsy used</u>

<u>Has your child ever had a status epilepticus (an epileptic seizure of more than 30 minutes)</u>

Behaviour

Behavioural diagnosis

Was there a behavioural diagnosis made by a (child) psychiatrist or psychologist?

Please specify

Does your child have a mood disorder

Please specify

Sleeping problems

Does your child have problems with sleeping

What kind of sleeping problems

Growth measurements

Birth measurements

Weight at birth (grams, ounces)

Length at birth (cm, inches)

Head circumference at birth (xx.x cm, inches)

Current measurements

Height (cm, feet, inches)

Weight (kg, pounds, ounces)

Head circumference (xx.x cm, inches)

Development

IQ

Is there developmental delay

Has an IQ, DQ or developmental test been done

What was the result of the testing

Milestones[a]

Gross motor milestones

Reach for objects

Since what age (months)

Roll over from lying on back to lying on tummy

Since what age (months)

Sit up unsupported

Since what age (months)

Get to a sitting position without help

Since what age (months)

Crawl or bottom shuffle

Since what age (months)

Pull him- or herself up a standing position

Since what age (months)

Walk holding on to furniture (e.g. to a table)

Since what age (months)

Walk unsupported

Since what age (months)

Fine motor milestones

Pick up something, like an object the size of a currant, between thumb and index finger

Since what age (months)

Hold a pencil between thumb and index finger

Since what age (months)

Colour within the lines

Since what age (months)

Build a tower of 6 blocks

Since what age (months)

String beads

Since what age (months)

Communication milestones

Smile

Since what age (weeks)

Make sounds: cooing, gurgle

Since what age (weeks)

Make sounds: babble, copy sounds he or she hears

Since what age (months)

Speak his or her first words

Since what age (months)

Speak 2-word sentences

Since what age (months)

Toilet training milestones

Is your child fully toilet trained (urine and defecation) during the day

Since what age (years)

Is your child fully toilet trained (urine and defecation) during the night

Since what age (years)

Adult functioning[b]

Ability to use telephone

Shopping

Food preparation

Housekeeping

Laundry

Mode of transportation

Responsibility for own medications

Ability to handle finances

[a] Developmental milestones were derived from the 'Van Wiechenschema', a developmental scale for children used by most Dutch health professionals[1].
[b] Questions on adult functioning were derived from the Lawton Instrumental Activities of Daily Living (IADL) Scale[2].

References

1. Nederlands Jeugdinstituut (NJi). Kennis: databank Instrumenten en richtlijnen: Van WiechenOnderzoek (VWO) [Internet]. Available from: https://www.nji.nl/instrumenten/van-wiechenonderzoek-vwo

2. Lawton MP, Brody EM. Assessment of older people: self-maintaining and instrumental activities of daily living. The Gerontologist. 1969;9(3):179–86.
